# Contact precautions for MRSA and VRE: where are we now? A survey of the *Society for Healthcare Epidemiology of America* Research Network

**DOI:** 10.1017/ash.2024.350

**Published:** 2024-09-20

**Authors:** Elise Martin, Daniel J. Morgan, Rachel Pryor, Gonzalo Bearman

**Affiliations:** 1 Department of Medicine, Division of Infectious Diseases, University of Pittsburgh School of Medicine, Pittsburgh, PA, USA; 2 Department of Epidemiology and Public Health, University of Maryland School of Medicine, Baltimore, MD, USA; 3 Quality and Safety Department, Virginia Commonwealth University Health Systems, Richmond, VA, USA; 4 Division of Infectious Diseases, Department of Medicine, Virginia Commonwealth University, Richmond, VA, USA

## Abstract

**Objective:**

Contact precautions for methicillin-resistant Staphylococcus aureus (MRSA) and vancomycin-resistant *Enterococcus* (VRE) have limited data on efficacy and have been associated with patient harm. Still, a 2015 *Society for Healthcare Epidemiology of America* (SHEA) Research Network (SRN) survey showed only 7% of hospitals discontinued routine MRSA/VRE contact precautions. The study objectives were to identify the current proportion of hospitals that have discontinued routine MRSA/VRE contact precautions and motivations for change.

**Design:**

An online survey was conducted of the SRN on current use and views of contact precautions for MRSA/VRE in each facility. An initial survey followed by 2 reminders was sent between 5/18/2021 and 6/9/2021.

**Participants:**

SRN facilities.

**Results:**

The response rate was 43% (37/87) of facilities surveyed and 35% of respondents were not routinely using contact precautions for MRSA and VRE. The most frequently reported reason for discontinuing contact precautions was research on the safety of discontinuing contact precautions without an increase in healthcare-associated infections (reported for 92% of facilities for MRSA and 100% for VRE). Of those using contact precautions, the most frequently reported reason to continue was a lack of safety data for discontinuation (MRSA 58% and VRE 46%). Most of those continuing contact precautions were interested in using contact precautions differently in their facility (MRSA 63% and VRE 58%).

**Conclusions:**

Over one in three healthcare facilities surveyed do not use contact precautions for MRSA or VRE. Most facilities choosing to continue contact precautions are interested in a different implementation strategy.

## Introduction

Contact precautions are a long-standing practice in acute care hospitals based on the theoretical mechanisms of methicillin-resistant *Staphylococcus aureus* (MRSA) and other multidrug-resistant organisms (MDRO) transfer and involve isolating patients and requiring gown and glove use. Although common and recommended by the Center for Disease Control, use of contact precautions for MRSA and vancomycin-resistant *Enterococcus* (VRE) is increasingly controversial given limited efficacy data and potential for patient harms.^1–4^ A survey of the *Society for Healthcare Epidemiology of America* (SHEA) Research Network (SRN), a consortium of acute care healthcare facilities, found that 93% hospitals used contact precautions for endemic MRSA and VRE in 2015.^1^ Despite widespread use, 63% of facilities were interested in using contact precautions differently, and most respondents cited concerns with decreased healthcare worker (HCW) visits and negative impacts on patient mental health and satisfaction. Only 38% of respondents were concerned that contact precautions increased adverse events.

Since that SRN survey, there have been multiple publications on the impacts of discontinuing contact precautions in acute care facilities. Although clinical trial data comparing contact precautions to standard precautions are limited, several quasi-experimental and interrupted time series analyses have demonstrated that contact precautions can be removed safely without an increase in MRSA and VRE healthcare-associated infections (HAIs), colonization, and device-associated infections.^5–11^ Most publications are from large, academic hospitals, with strong horizontal infection prevention practices and good hand hygiene. One study from a large health system found that a variety of facility types could safely remove MRSA/VRE contact precautions as long as MRSA/VRE rates were low and hand hygiene compliance was high.^11^

Several studies explored the possible connection of contact precautions to noninfectious patient harms, including delays in admission and discharge, decreased HCW contact, chart documentation gaps, new onset anxiety and depression, poor satisfaction, and increased preventable noninfectious adverse events.^12–24^ One health system found a reduction in noninfectious adverse events after contact precautions were discontinued and noted that the patients with MRSA/VRE who were no longer in isolation had the largest reduction, with a 72% reduction in noninfectious adverse events after contact precautions were discontinued.^25^

Despite recent data on safety, SHEA, Infectious Diseases Society of America (IDSA), the Association for Professionals in Infection Control and Epidemiology (APIC), the American Hospital Association (AHA), and The Joint Commission still considered contact precautions for MRSA an “essential practice” in the 2022 Compendium, although they noted a facility could chose to forgo contact precautions after performing an MRSA risk assessment, use of other mitigating strategies, and ongoing monitoring and oversight.^26^ Additionally, many facilities continue contact precautions given potential benefit, although these publications use a combination of strategies and it is difficult to tease out which of the bundled approaches led to decreased transmission.^1,27^

Given ongoing controversy on contact precautions for MRSA/VRE as well as updated risk/benefit data, an independent group of healthcare epidemiologists conducted a repeat survey of healthcare facilities within the SRN to determine if, and to what extent, contact precautions practices changed since the previous survey published in 2015.^1^

## Methods

SRN facilities collaborate on multicenter research projects in healthcare epidemiology and antimicrobial stewardship.^28^ One principal investigator per facility can join on behalf of their facility and participate in projects. All facilities active in SRN in 2021 were invited via e-mail to participate in the 26-question survey on the use of contact precautions for MDROs in their facility. The survey was administered between 5/18/2021 and 6/9/2021. An initial survey was sent with 2 reminder e-mails. Unanswered questions were not included in analysis. Survey responses were primarily categorical and Likert scale. Descriptive analyses were performed using Microsoft Excel software (Microsoft, Redmond, WA). This survey was approved by the University of Pittsburgh institutional review board.

## Results

Of the 87 facilities actively participating in SRN in 2021, 37 institutions responded to the SRN survey (43% response rate). Respondents included healthcare epidemiologists, infection committee chairs, clinical and basic science researchers, patient care providers, teaching/educators, administrators, clinical microbiologists, public health providers, quality and safety chairs, and infection preventionists. Fifty-nine percent of responses came from academic medical centers, 16% from community teaching hospitals with academic affiliation, 8% from nonacademic community hospitals, 8% from public hospitals, and 8% other. Facilities ranged from less than 100 beds to over 1,000 beds, and most included adult and pediatric populations.

**Figure 1.**
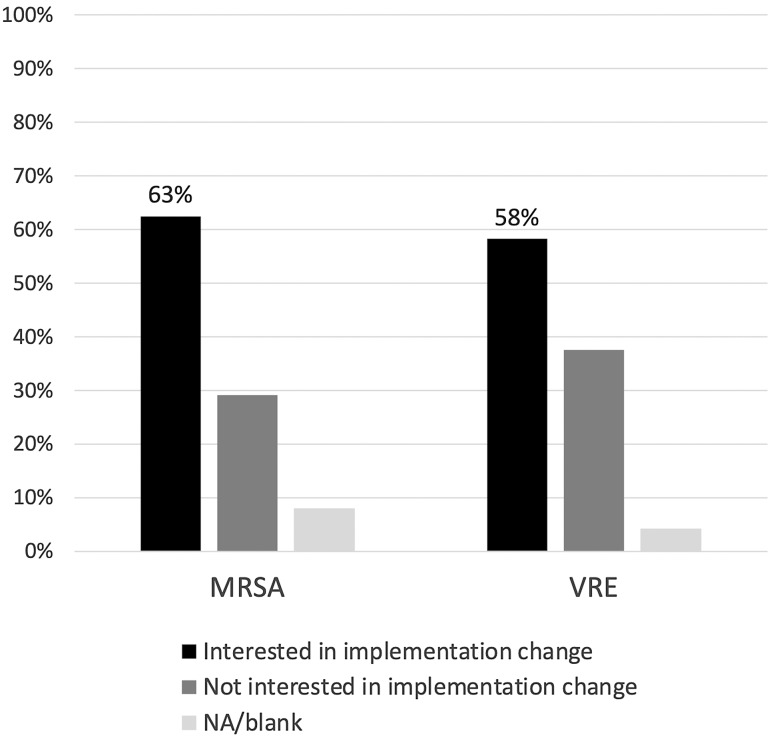
Current interest in applying contact precautions differently among facilities still using contact precautions for MRSA and/or VRE.

Of the facilities who responded, 35% reported not using contact precautions for MRSA and 35% not using contact precautions for VRE at the time of the survey (Table 1). For extended spectrum beta-lactamase (ESBL) organisms, 30% of facilities did not use contact precautions, and no facilities reported discontinuing contact precautions for carbapenem-resistant *Enterobacterales* (CRE). Of the facilities not using contact precautions for MRSA, 46% discontinued contact precautions over 6 years prior to the survey and 38% had discontinued MRSA precautions within the last 6 years. For VRE, 46% discontinued over 6 years ago and 31% had discontinued precautions within the last 6 years. Although the survey was conducted during the COVID-19 pandemic, only 15–23% of those not isolating for MRSA and/or VRE made the change in response to personal protective equipment (PPE) conservation for COVID-19. Of the 24 facilities still using contact precautions for MRSA and VRE, 63% were interested in using contact precautions differently for MRSA and 58% were interested in using contact precautions differently for VRE (Figure 1).

**Table 1.** Survey responses to current use of contact precautions in their facility

Discontinued contact precautions (n = 37)	% of facilities (n)
MRSA	35% (13)
VRE	35% (13)
ESBL	30% (11)
CRE	0% (0)
**Timing of discontinuation—MRSA (n = 13)**	
Within the past 6 years	38% (5)
More than 6 years ago	46% (6)
During COVID-19 only	15% (2)
**Timing of discontinuation—VRE (n = 13)**	
Within the past 6 years	46% (6)
More than 6 years ago	31% (4)
During COVID-19 only	23% (3)
**Data used to determine isolation (n = 26)**	
Clinical isolates	77% (20)
Active surveillance tests	77% (20)
Known history of the organism	46% (12)
Other	4% (1)
**Frequency of combining colonized and non-colonized patients in multi-occupancy rooms (n = 37)**	
Always	5% (2)
Frequently	14% (5)
Only when necessary due to bed shortage	24% (9)
Never	24% (9)
Not applicable (only single rooms)	19% (7)
Other	14% (5)

Most facilities based isolation status on clinical isolates (77%) and active surveillance cultures (77%), with only 46% basing isolation status on known history of an organism (Table 1). In double or multi-occupancy rooms, 19% frequently or always isolated non-colonized patients with patients known to be colonized with MRSA and/or VRE, while 24% only do so in times of bed shortage, and 24% never co-room colonized and non-colonized patients together (Table 1).

**Figure 2.**
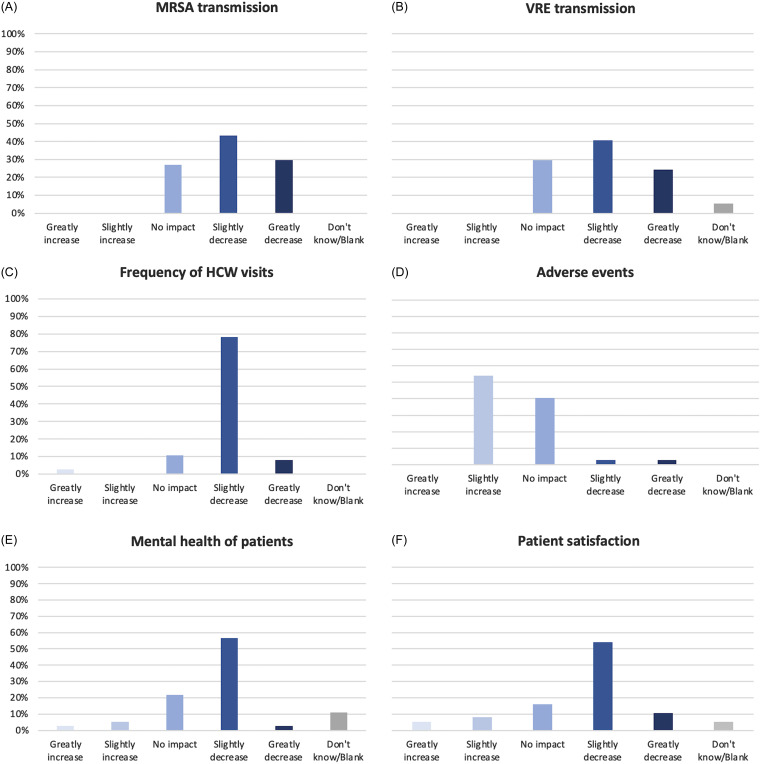
Survey respondents’ beliefs on the impact of contact precautions on transmission and potential patient harms.

Respondents were asked about their opinions on the impact of contact precautions on transmission and potential patient harms (Figure 2). Most respondents (73%) believed that contact precautions either slightly or greatly reduced MRSA transmission, with 27% believing there was no impact, and no respondents believed they increased MRSA transmission. Similarly, 65% of respondents believed that contact precautions either slightly or greatly reduced VRE transmission, with 27% believing there was no impact, and no respondents believed they increased VRE transmission. Most survey respondents (54%) believed that contact precautions slightly increase adverse events, while 41% believed that had no impact. Six percent thought that they would decrease adverse events. Seventy-eight percent of those surveyed were concerned that contact precautions can lead to a decrease in HCW visits, with an additional 8% believing that they greatly decrease HCW visits. When asked about mental health impacts, 57% of respondents were concerned that they would slightly worsen mental health, with an additional 3% believing they greatly worsen mental health. Twenty-two percent did not think there would be an impact and only 8% thought they could improve mental health. Similarly, most respondents thought contact precautions slightly (54%) or greatly (11%) decrease patient satisfaction. Only 16% believed contact precautions would not impact, and 13% believed it could improve satisfaction.

When asked about the views of HCW in their facilities, 70% of respondents believed that their HCW consider contact precautions a hassle. Despite this, only 35% thought HCW believe they should be used less often. Only 24% thought HCW believe they benefit patients, while only 11% think that HCW would feel contact precautions adversely affect patients. Eight percent thought HCW think contact precautions should be used more. Sixty-five percent thought the response would vary by unit and 16% thought they would have no preference.

**Figure 3.**
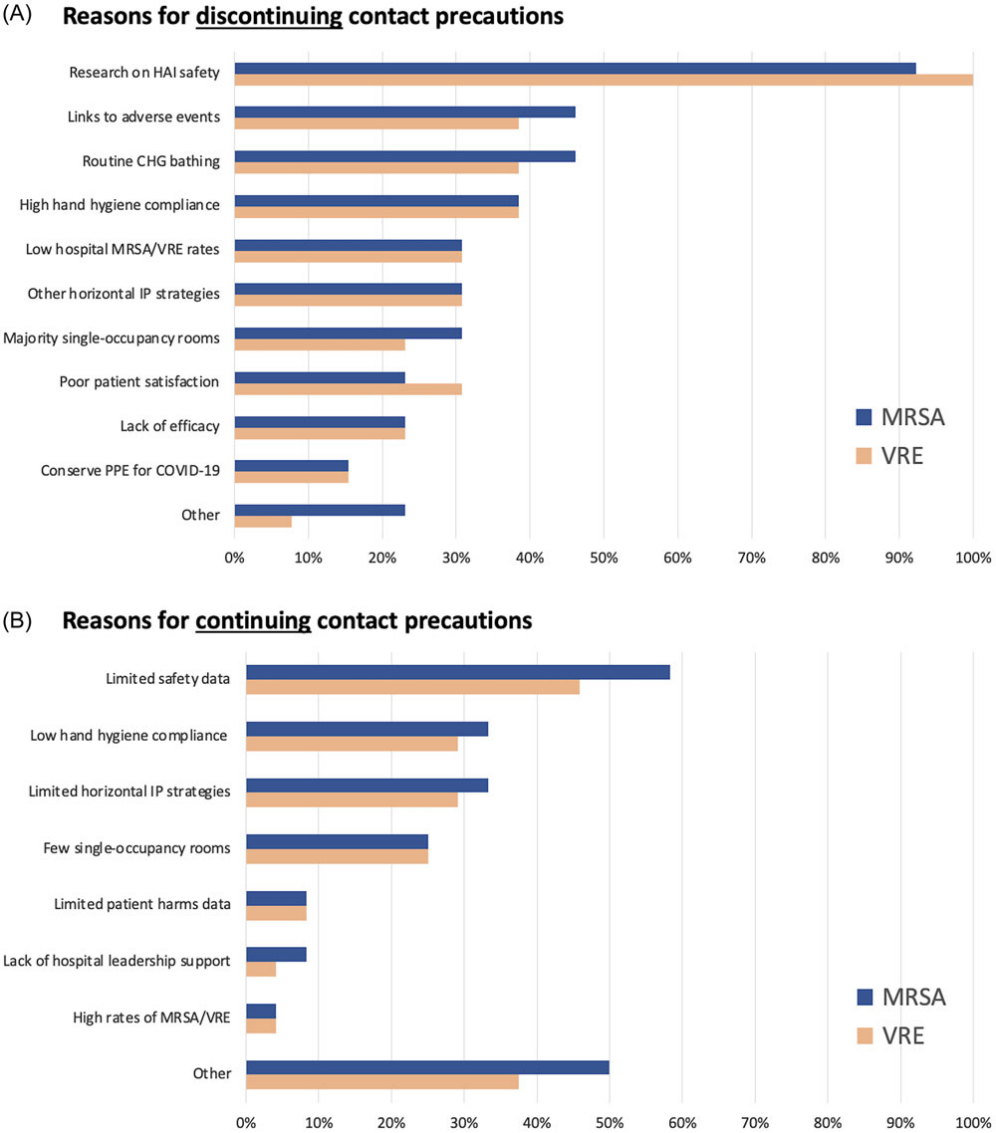
Institutional motivations for why institutions discontinued or are currently using contact precautions for MRSA and/or VRE.

Among facilities that discontinued contact precautions for MRSA and VRE, 92–100% of respondents reported that research findings that contact precautions can be discontinued without an increase in HAI led to their institutions’ decision to discontinue contact precautions (Figure 3). Although no other reason had a majority, other common responses included links to adverse events (MRSA 46% and VRE 38%), use of routine chlorhexidine gluconate bathing (46% and 38%), high hand hygiene compliance (38%), low hospital MRSA/VRE rates (31%), use of other horizontal infection prevention strategies (31%), majority single occupancy rooms in their facility (31%, 21%), and poor patient satisfaction (23%, 31%). Only 23% cited a lack of efficacy and 15% cited the need to conserve PPE for the COVID-19 pandemic. Other responses included a lack of single occupancy rooms for isolation and impact of contact precautions on patient flow in the hospital.

Among facilities that are continuing contact precautions for MRSA (n = 24) and/or VRE (n = 24), the most common response was limited safety data for discontinuation of contact precautions for MRSA (58%) or VRE (46%). Although no other reason had a majority, other common responses included low facility hand hygiene compliance (MRSA 33% and VRE 29%), limited use of other horizontal infection prevention strategies, (33% and 29%), and few single occupancy rooms (25%). Fifty percent of those continuing contact precautions for MRSA selected “other” and these responses included concern for adherence to standard precautions with uncontrolled secretions, federal Veterans Affairs mandate, and desire to keep MRSA cases low. Five (21%) wrote in a response that contact precautions are an effective preventative strategy based on literature or internal facility experience. Thirty-eight percent of respondents selected other as a motivation to continue VRE precautions, and their responses included literature or facility evidence of benefit (17%), desire to keep VRE cases low, and lack of capacity to initiate discontinuation.

## Discussion

From 2015 to 2021, the discontinuation of routine contact precautions for endemic MRSA and VRE has become more common. In 2015, only 7% of SRN hospitals reported that they were not using routine contact precautions^1^, and this has increased to 35% based on this survey. Views on negative impacts of contact precautions are also shifting. In 2015, 78% of respondents believed that contact precautions lead to decreased HCW visits, and this number has risen to 86%. Most respondents remained concerned with the impacts on mental health and patient satisfaction, but concern for the negative impacts on mental health decreased slightly from 68% to 60% and concern for the negative impacts on patient satisfaction decreased from 69% to 65%. Interestingly, the concern for adverse events increased from only 38% of respondents in 2015 to 54% in 2021 and was a common motivating factor for the facilities that removed routine contact precautions.

Since the last SRN survey on contact precautions, multiple quasi-experimental and interrupted time series analyses demonstrated that contact precautions were discontinued without an increase in MRSA/VRE HAI and device-associated infections, as long as other horizontal infection prevention strategies are in use.^5–11^ The facilities in this survey that reported discontinuing contact precautions cited that research as the most common factor motivating their institutions decision to discontinue contact precautions (>90%). When asked about other horizontal infection prevention strategies and current MRSA rates, each of the results were a minority. Thus, no specific alternative factor was the main diver, and this differed based on facility. Interestingly, approximately half of those that removed precautions for MRSA and VRE did so, since the last SRN survey and as the recent studies on safety were published.

Of the facilities that did not discontinue precautions, there were a variety of reasons that led to the decision to continue contact precautions. Only half of facilities sited lack of safety data and others did state that they believed that contact precautions were effective to reduce transmission. Despite this, 58–63% of the facilities that are still using contact precautions are interested in implementing them differently. Further research on what additional aspects of safety data are necessarily for these sites would be helpful as guidance on MRSA/VRE prevention strategies are developed. Additionally, further research on other ways to implement contact precautions, including risk-stratified approaches focused on high-risk locations and/or high-risk HCW, could provide some transmission protection while avoiding the complete discontinuation that some facilities are resistant to.

Although this SRN survey provides valuable information on the changing views of the SRN sites, the response rate to this survey was low and may not reflect the views of hospitals not involved in SRN or those SRN facilities that did not respond to the survey. The language of the survey was used or briefly summarized in the results. Given this survey incorporates the participants beliefs and motivations, the language of the survey could have impacted the results. This survey was conducted in 2021, during the COVID-19 pandemic, which possibly contributed to the low response rate. This survey was also conducted prior to the release of SHEA compendium on MRSA^26^, and the current rates of routine contact precautions may have changed.

Contact precautions for MRSA and VRE remain controversial, but newer data suggest that contact precautions can be safely discontinued without an increase in MRSA/VRE HAIs. The SRN membership includes an increasing number of facilities who discontinued routine MRSA/VRE contact precautions in response to this new data, although most facilities still employ contact precautions for MRSA and VRE. Further research on safety of discontinuing contact precautions is still necessary, as well as alternative strategies for implementing contact precautions and ways to mitigate patient harms in locations that still use them.
